# Bacteriological Profile and Predictors of Death Among Neonates With Blood Culture-Proven Sepsis in a National Hospital in Tanzania—A Retrospective Cohort Study

**DOI:** 10.3389/fped.2022.797208

**Published:** 2022-04-05

**Authors:** Nour Abdallah Ba-alwi, John Ogooluwa Aremu, Michael Ntim, Russel Takam, Mwanaidi Amiri Msuya, Hamid Nassor, Hong Ji

**Affiliations:** ^1^Department of Pediatrics, First Affiliated Hospital of Dalian Medical University, Dalian, China; ^2^Department of Anatomy, Harbin Medical University, Harbin, China; ^3^Department of Physiology, Kwame Nkrumah University of Science and Technology, Kumasi, Ghana; ^4^Hei Kaola Pediatric Clinic, Dalian, China; ^5^Muhimbili National Hospital, Dar es Salaam, Tanzania; ^6^Temeke Regional Referral Hospital, Dar es Salaam, Tanzania

**Keywords:** neonatal sepsis, sepsis, bacteriological profile, antibiotic susceptibility, Gram-negative bacteria, Gram-positive bacteria

## Abstract

**Background:**

Neonatal sepsis is still a major cause of death and morbidity in newborns all over the world. Despite substantial developments in diagnosis, treatments, and prevention strategies, sepsis remains a common problem in clinical practice, particularly in low-resource countries.

**Methods:**

A retrospective cohort study of 238 neonates with positive blood culture-proven sepsis (in Muhimbili National Hospital) was conducted from January 2019 to December 2020. The outcomes of hospitalization were survival and death.

**Results:**

In total, 45.4% mortality resulted from 238 neonates who had sepsis exclusively based on blood culture positivity. A significant association was found between very low birth weight (VLBW), hyperglycemia, mechanical ventilation, and high neonatal mortality. Among the different clinical presentations of neonatal sepsis, lethargy, vomiting, and respiratory distress were found to be frequently associated with neonatal mortality. Furthermore, sepsis with Gram-negative bacteria and early-onset sepsis were also associated with high neonatal mortality. Of the 108 neonatal deaths, the largest proportion (40%) was observed with *Staphylococcus aureus*, and the remaining 38% was caused by *Klebsiella*, 14% by *Escherichia coli*, 5% by *Pseudomonas*, 4% by *Acinetobacter*, and 2% by *Streptococcus*. No neonatal deaths from *Serratia* infection were observed. The overall resistance of isolated organisms to the recommended first-line antibiotics was 84% for ampicillin and 71.3% for gentamicin. The resistance pattern for the recommended second-line antibiotics was 76.2% for ceftriaxone, 35.9% for vancomycin, and 17.5% for amikacin.

**Conclusion:**

VLBW, early-onset sepsis, clinical and laboratory parameters like lethargy, vomiting, and hyperglycemia, sepsis with Gram-negative bacteria, and being on mechanical ventilation are strong predictors of death in neonatal sepsis. In addition, this study discovered extraordinarily high resistance to conventional antibiotics. These findings give light on the crucial aspects to consider in preventing this disease and poor outcomes.

## Introduction

Neonatal sepsis is a clinical condition defined as an infection in newborns accompanied by or caused by an infection of the blood, typically bacterial and rarely fungal. Its symptoms and clinical features include temperature instability, respiratory difficulties, and refusal to eat (1). Neonatal sepsis can be categorized as early-onset sepsis (EOS) and late-onset sepsis (LOS). EOS is defined as the onset of signs and symptoms of infection within 72 h of life and may be associated with pathogen isolation or not. In LOS, signs and symptoms are present after 72 h of life (2). Sepsis is the most common reason for infant mortality, with around 2.4 million fatalities each year or 6,700 per day (3). Neonatal deaths that occur around the world each year total to ~4 million. About 30–50% of the total newborn fatalities in underdeveloped nations are caused by neonatal sepsis (4). Early detection, appropriate antibiotic medication, and aggressive supportive care are all elements of prompt diagnosis (4). The risk of newborn death is greatest during the first 28 days of life, and children below 5 years are most vulnerable to sepsis. According to the findings of a new study, more than three-quarters of neonatal deaths occur in the first week of life, with one-third of all neonatal deaths occurring on the first day after birth (5).

Antimicrobial resistance is a major factor in the development of sepsis and septic shock. Every year, over 214,000 newborns die from sepsis caused by resistant infections (6). Neonatal sepsis is a serious global health issue, resulting in significant health burdens due to medical expenses and productivity loss as well as negative consequences on health and quality of life. Africa experiences the slowest decrease in mortality globally, where neonatal mortality decrease has been slower compared to maternal and child mortality reductions—with stillbirth rates being the slowest (7). Neonatal fatalities occur quickly, necessitating rapid medical interventions. According to a study conducted in Tanzania by Mangu et al. newborn fatalities accounted for 11.3% of all in-hospital deaths (8). Most neonatal deaths occur in the first week of life.

To ensure the efficient and long-term detection of newborn sepsis, there is a need to understand the diagnosis, etiology, and treatment of neonatal sepsis at all levels of the health system. The response to antimicrobial drugs may vary substantially over time and across regions, undermining the efficacy of empirical therapy (9).

This study was conducted to understand the bacteriological profile and predictors of death among neonates with blood culture-proven sepsis in Muhimbili National Hospital, Tanzania. This hospital is the national and tertiary referral hospital that receives sick neonates from different health facilities, whether private or public, across the country and is thus a critical spot to determine retrospectively the efficacy of medications provided in combating neonatal infections in the last 2 years.

## Materials and Methodology

### Study Design, Area, and Period

A retrospective cohort study of recorded data from hospital pediatric database and files was used to study neonates who were admitted to the neonatal department, diagnosed with sepsis, and had positive blood cultures. This study was conducted from January 2019 to December 2020 in the neonatal department of a tertiary referral and teaching hospital—Muhimbili National Hospital in Dar es Salaam, Tanzania. Patient medical records including clinical symptoms, hematological parameters, pathogen types, and antimicrobial susceptibility were reviewed. A data collection sheet was designed and used to obtain socio-demographic data and other relevant factors related to neonatal sepsis, like maternal fever, prolonged rupture of the membrane (PROM), mode of delivery, birth weight of the baby, gestational age (<37 completed weeks was considered as premature), the temperature of the infant, vomiting, respiratory rate, jaundice, umbilical redness, convulsions, and inability to breastfeed.

### Study Population

A total of 238 neonates with positive blood cultures were included in the study. The sample size was determined using the single-population proportion formula based on the prevalence of positive blood cultures of 19.2% as found by Mkony et al. (10) in the same hospital. Allowing 5% margin of error (MOE) with 95% confidence interval (CI), (*z*/2 = 1.96) using a single-population proportion (*p*) formula, 1 - *p* being the proportion of the population that does not possess the character of interest, the sample size was calculated as follows:

*n* = (Z_α/2_)^2^^*^p^*^(1-*p*)/MOE^2^

### Inclusion/Exclusion Criteria

All neonates who were admitted with blood culture positives within the specified time were included in this study. However, clinically suspected cases with negative blood cultures as well as neonates with positive blood cultures but with incomplete or missing records were excluded from this study. All neonates who died <3 days from birth were not included in the study. Blood cultures with *Staphylococcus* coagulase-negative as well as yeast infections were excluded from the study.

### Data Collection

The primary researcher collected information from the hospital's pediatric database and files on all positive blood culture-proved sepsis cases admitted between January 2019 and December 2020. The medical files were traced using the patients' card number on the registry. Data collection sheets were employed; the data sheets were filled after reading through the manually filled files containing written histories and investigations performed by patients during their stay. Age, sex, birth weight, gestational age at birth (term or preterm), Apgar score at 5 min, place of delivery, mode of delivery, and specific clinical features, such as jaundice, temperature instability (hypothermia, hyperthermia), respiratory distress, poor feeding, vomiting, convulsions, poor reflexes, pallor, jaundice, and umbilical redness, are among the neonatal data collected. Maternal obstetric history of PROM that lasted more than 24 h, maternal urinary tract infection, antibiotic usage, and presence of chorioamnionitis were all noted. Blood culture profile, hemoglobin levels, random blood glucose (RGB) levels, white blood cells, platelets, and C-reactive protein (CRP) levels were among the laboratory features investigated. Anemia was defined as hemoglobin level <10 g/dl, while leucopenia was defined as total white blood count <5,000/mm^3^, leukocytosis was defined as total white blood count >20,000/mm, and thrombocytopenia was defined as a platelet count of <150,000/mm^2^. According to the Muhimbili National Hospital, CRP was considered positive when it rises above 5 mg/L, while in other studies CRP is considered positive when it is elevated above 10 mg/L (11). Hospitalization outcomes were documented as survival at discharge and at death. Any neonate who died 3 days or more from birth at the neonatal unit was considered deceased (mortality). The total sample size of neonates determined was then divided into two groups. Group A consisted of neonates who lived until discharge, while Group B consisted of neonates who died while in the hospital.

### Blood Culture Specimen Collection

Under aseptic conditions, each study patient has two blood specimens that were taken for the blood cultures from different peripheral venipuncture sites at 1/2- to 1-h intervals by the hospital protocol; however, the times were not documented. Approximately, 2–5 ml of blood was drawn and kept in aerobic culture bottles, and the sample was immediately transported to the Central Pathology Laboratory at Muhimbili National Hospital (MNH) for processing. Antimicrobial sensitivity testing was carried out at MNH for ampicillin, cloxacillin, and gentamicin, which are used as first-line antibiotics, as well as ceftriaxone, vancomycin, and amikacin, which are used as second-line medications for the treatment of newborn sepsis. The results were classified as resistant, intermediate, or sensitive. During data analysis, isolates with intermediate resistance were labeled as resistant.

### Statistical Analysis

The extracted data was processed using SPSS 20.0 software by performing descriptive and inferential statistics. Student's *t*-test was used to compare the means. Statistical significance was established if the *p*-value was <0.05 at 95% CI. The parameters that had significant correlations with death were considered potential risk factors for a poor outcome in newborn sepsis. These variables were used to get the odds ratio by using risk estimate analysis.

### Ethical Consideration

The study was approved by the ethics committee of Muhimbili National Hospital, Tanzania.

## Results

### Baseline Characteristics

Out of a total of 238 newborns with positive blood cultures (128 male and 110 female), 108 died (57 male, 51 female), accounting for a mortality rate of 45.4% (44.5% male, 46.4% female) of the neonates with blood culture-proven sepsis. Table 1 shows that the case fatality rate for early-onset sepsis was 55.9% (61 out of 109), which was higher than the 36.4% rate for late-onset sepsis (47 out of 129). The case fatality rate was 66.7% in infants whose mothers had chorioamnionitis (10 out of 15 neonates). Furthermore, neonates with birth weights of <1.5 kg (54.8%), preterm infants (49.0%), and those delivered at home (63.6%) had a higher case fatality rate than those with birth weights >1.5 kg (43.6%), term infants (39.1%), and those delivered at a health facility (44.5%), respectively. In addition, newborns whose mothers had PROM for more than 24 h had a 60% case fatality rate (30 out of 50 neonates). There was a reduction in case fatalities in neonates whose mothers utilized antibiotics during pregnancy (31.2%).

**Table 1:** Distribution of case fatality rate (CFR) of 238 study neonates with culture-proven sepsis based on infant and maternal factors.

**Characteristics**	**Number (*n*)**	**Number of deaths (CFR%)**
**Age**		
≤ 72 h	109	61 (56.0)
>72 h	129	47 (36.4)
**Sex**		
Male	128	57 (44.5)
Female	110	51 (46.4)
**Body weight, kg**		
<1.5	73	40 (54.8)
1.5–2.5	94	41 (43.6)
≥2.5	71	27 (38.0)
**Gestational age**		
Term, >37 weeks	87	34 (39.1)
Preterm, <37 weeks	151	74 (49.0)
**Place of birth**		
Health facility	227	101 (44.5)
Home	11	7 (63.6)
**Mode of delivery**		
SVD	162	81 (50.0)
C/S	76	27 (35.5)
**Apgar score at 5 min**		
<7	41	19 (46.3)
>7	197	89 (45.2)
**PROM**		
<24 h	180	78 (43.3)
>24 h	58	30 (51.7)
**Maternal UTI**		
Yes	22	6 (27.3)
No	216	102 (47.2)
**Antibiotic use**		
Yes	16	5 (31.2)
No	222	103 (46.4)
**Chorioamnionitis**		
Yes	15	10 (66.7)
No	223	98 (43.9)

*PROM, prolonged rupture of membranes; UTI, urinary tract infection*.

Table 2 shows that infants with vomiting (63.2%), lethargy (59.1%), convulsions (62.5%), and respiratory distress (53.5%) died at a higher rate than those with other clinical characteristics. Random blood glucose (RBG) levels (65.5%) as well as leukocytosis (62.5%) were the laboratory indicators related to a higher frequency of newborn deaths due to neonatal sepsis. There was no significant difference between neonates with positive CRP, thrombocytopenia, and leukopenia who survived or died due to neonatal sepsis.

**Table 2:** Comparison of neonates with sepsis between those who survived and those who died according to clinical features and laboratory parameters.

**Parameter**	**Alive (*N*)**	**Deceased (*N*)**	**Alive (%)**	**Deceased (%)**	***P*-value**
**Poor feeding**					
Yes	29	29	50	50	0.418
No	101	79	56.1	43.9	
**Lethargy**					
Yes	36	52	40.9	59.1	0.001
No	94	56	62.7	37.3	
**Vomiting**					
Yes	28	48	36.8	63.2	0.001
No	102	60	63.0	37.0	
**Convulsions**					
Yes	9	15	37.5	62.5	0.085
No	121	93	56.5	43.5	
**Temperature instability**					
Yes	95	78	54.9	45.1	0.883
No	35	30	53.9	46.1	
**Pallor**					
Yes	38	39	49.4	50.6	0.278
No	91	69	56.9	43.1	
**Jaundice**					
Yes	83	64	56.5	43.5	0.471
No	47	44	51.7	48.3	
**Respiratory distress**					
Yes	80	92	46.5	53.5	0.001
No	50	16	75.8	24.2	
**Umbilical redness**					
Yes	18	10	64.3	35.7	0.268
No	112	98	53.3	46.7	
**Thrombocytopenia**					
≤ 150,000/ul, yes	58	55	51.3	48.7	0.334
>150,000/ul, no	72	53	57.6	42.4	
**Leukopenia**					
≤ 5,000/ul, yes	39	22	63.9	36.1	0.087
>5,000/ul, no	91	86	51.4	48.6	
**Leukocytosis**					
>20,000/ul, yes	9	15	37.5	62.5	0.085
≤ 20,000/ul, no	121	93	56.5	43.5	
**Hb level**					
≤ 10 g/dl	30	21	58.8	41.2	0.449
>10 g/dl	100	87	53.5	46.5	
**RBG level**					
>6.9 mmol/l, high	10	19	34.5	65.5	0.004
<3.5 mmol/l, low	21	25	45.7	54.3	
3.5–6.9 mmol/l, normal	99	64	60.7	39.3	
**CRP**					
>5 mg/L, positive	77	75	50.7	49.3	0.101
≤ 5 mg/L, negative	53	33	61.6	38.4	

### Associating Factors for Death Among Septicemic Babies

All the variables in the study were subjected to odds ratio analysis to determine whether variables had a positive correlation with neonatal death or poor prognosis among neonates with sepsis. The results of the analysis are provided in Table 3. It was observed that the following factors were all significant in determining their association to death in neonates with sepsis: very low birth weight (OR, 1.441), early-onset sepsis (OR, 1.536), vomiting (OR, 1.705), lethargy (OR, 1.583), respiratory distress (OR, 2.206), high RBG level (OR, 1.669), positive blood culture for Gram-negative bacteria (OR, 1.424), and being on mechanical ventilation (OR, 2.206).

**Table 3:** Risk estimate analysis of the association factors to death among septicemic babies.

	**Number**		**Percent**			**Deceased**	
**Parameter**	**Alive**	**Deceased**	**Alive**	**Deceased**	***p*-values**	**Odds ratio**	**95% confidence interval**
**Age**							
≤ 72 h	48	61	44.0	56.0	0.002	1.536	(1.158–2.037)
>72 h	82	47	63.6	36.4			
**Mode of delivery**							
VD	81	81	50	50	0.035	1.407	(1.002–1.977)
CS	49	27	64.5	35.5			
**Birth weight**							
<1.5 kg	33	40	45.2	54.8	0.044	1.441	(1.003–2.071)
>2.5 kg	44	27	62.0	38.0			
**Lethargy**							
Yes	36	52	40.9	59.1	0.001	1.583	(1.208–2.075)
No	94	56	62.7	37.3			
**Vomiting**							
Yes	28	48	36.8	63.2	0.001	1.705	(1.309–2.221)
No	102	60	63.0	37.0			
**Respiratory distress**							
Yes	80	92	46.5	53.5	0.001	2.206	(1.409–3.456)
No	50	16	75.8	24.2			
**RBG level**							
>6.9 mmol/l, high	10	19	34.5	65.5	0.009	1.669	(1.205–2.311)
3.5–6.9 mmol/l, normal	99	64	60.7	39.3			
**Mechanical ventilation**							
Yes	3	23	11.5	88.5	0.001	2.206	(1.779–2.939)
No	127	85	59.9	40.1			
**Gram-negative bacteria**							
Yes	55	63	46.6	53.4	0.014	1.424	(1.07–1.895)
No	75	45	62.5	37.5			

*VD, vaginal delivery; C/S, cesarean section; RBG, random blood glucose*.

### Causative Bacteria of Neonatal Sepsis

**Figure 1:**
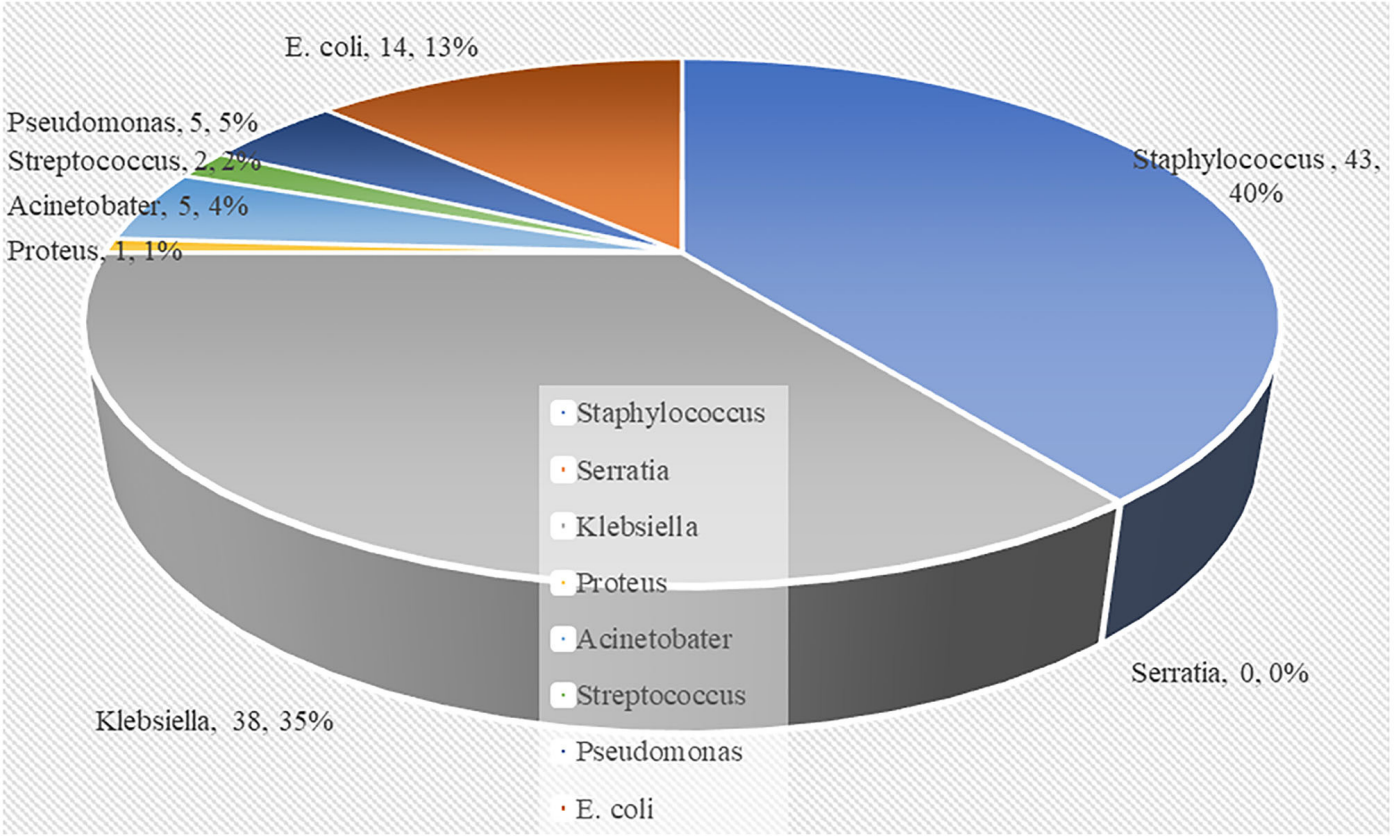
Pie chart showing the distribution of deceased neonates according to the causative bacteria.

According to the results in Table 4, Gram-positive bacteria (120 out of 238 neonates) predominated in causing newborn sepsis as compared to Gram-negative bacteria (118 out of 238 neonates). Gram-negative bacteria (53.8%) outnumbered Gram-positive bacteria in early-onset sepsis. In late-onset sepsis, Gram-positive bacteria predominated (60.0%) over Gram-negative bacteria. *Klebsiella* species (56.2%), *Escherichia coli, Staphylococcus aureus*, and *Pseudomonas* species were shown to predominate in early-onset newborn sepsis. *S. aureus* is the most common cause of late-onset sepsis. Figure 1 shows that sepsis caused by Gram-negative bacteria was related to a higher proportion of neonate mortality than Gram-positive bacteria. Of the 108 neonatal deaths, the largest proportion (40%) was observed with *S. aureus*, and the remaining 38% was caused by *Klebsiella* species, 14% by *E. coli*, 5% by *Pseudomonas* species, 4% by *Acinetobacter* species, and 2% by *Streptococcus* species. No neonatal deaths from *Serratia* species infection were observed.

**Table 4:** Distribution of microorganisms according to time of infection.

	**EOS, *N* (%)**	**LOS, *N* (%)**	**Total**	***P*-value**
**Gram-positive**
*Staphylococcus aureus*	48 (41.0)	69 (59.0)	117	0.089
*Streptococcus* spp.	0	3 (100)	3	0.083
**Gram-negative**
*Klebsiella* spp.	41 (56.2)	32 (43.8)	73	0.071
*Pseudomonas* spp.	5 (71.4)	2 (28.6)	7	0.198
*Acinetobacter* spp.	3 (37.5)	5 (62.5)	8	0.6
*Escherichia coli*	13 (46.4)	15 (53.6)	28	0.981
*Serratia* spp.	0	1 (100)	1	0.351
*Proteus* spp.	1 (100)	0	1	0.32
Gram-positive	48 (40.0)	72 (60.0)	120	
Gram-negative	63 (53.8)	55 (46.3)	118	0.039

A greater proportion of neonates with Gram-positive infections were neonates with low birth weight. Majority of the neonates were *Staphylococcus*-infected and were mostly <2.5 kg at birth. A similar distribution was observed in the Gram-negative-infected neonates. *Klebsiella* infection was dominant among the neonates <2.5 kg (low birth weight) (Table 5). Gram-negative sepsis predominates in the preterm group (about 37.8%), while Gram-positive sepsis was at about 25.6%. However, when it comes to specific causative bacteria, we can see that *S. aureus* and *Klebsiella* are equally prevalent as a cause of neonatal sepsis in preterm infants with 24.4%. Gram-positive bacteria (24.8%) outnumber the Gram-negative bacteria (11.8%) in the term neonates. Furthermore, *S. aureus* had a higher percentage (24.8%) than *Klebsiella* spp. (6.3%). Overall, *S. aureus* was nearly equally responsible for sepsis in the preterm (24.4%) and term neonates (24.8%) (Table 6).

**Table 5:** Distribution of microorganisms according to birth weight classification.

	**Birth weight**, ***n*** **(%)**				
**Causative bacteria**	**<1.5 kg**	**1.5–2.5 kg**	**>2.5 kg**	**Total**	***P*-value**
**Gram-positive**	25 (10.5)	44 (18.5)	51 (21.4)	120	0
*Staphylococcus aureus*	24 (10.1)	44 (18.5)	49 (20.5)	117	0
*Streptococcus* spp.	1 (0.4)	0 (0)	2 (0.8)	3	0.2
**Gram-negative**	48 (20.2)	50 (21.0)	20 (8.4)	118	0
*Klebsiella* spp.	35 (14.7)	27 (11.3)	11 (4.6)	73	0
*E. coli*	9 (3.8)	13 (5.6)	6 (2.5)	28	0.56
*Acinetobacter* spp.	2 (0.8)	5 (2.1)	1 (0.4)	8	0.362
*Pseudomonas* spp.	2 (0.8)	3 (1.3)	2 (0.8)	7	0.983
*Proteus* spp.	0 (0)	1 (0.4)	0 (0)	1	0.463
*Serratia* spp.	0 (0)	1 (0.4)	0 (0)	1	0.463

**Table 6:** Distribution of microorganisms according to gestational age classification.

	**Gestational age**, ***n*** **(%)**			
**Causative bacteria**	**Pre-term**	**Term**	**Total**	***P*-value**
**Gram-positive**	61 (25.6)	59 (24.8)	120 (50.4)	0
*Staphylococcus aureus*	59 (24.4)	58 (24.8)	117 (49.2)	0
*Streptococcus* spp.	2 (0.8)	1 (0.4)	3 (1.3)	0.907
**Gram-negative**	90 (37.8)	28 (11.8)	118 (49.6)	0
*Klebsiella* spp.	58 (24.4)	15 (6.3)	73 (30.7)	0.001
*E. coli*	21 (8.8)	7 (2.9)	28 (11.8)	0.177
*Acinetobacter* spp.	6 (2.5)	2 (0.8)	8 (3.4)	0.490
*Pseudomonas* spp.	3 (1.3)	4 (1.7)	7 (2.9)	0.251
*Proteus* spp.	1 (0.4)	0 (0.0)	1 (0.4)	0.447
*Serratia* spp.	1 (0.4)	0 (0.0)	1 (0.4)	0.447

### Antibiotic Susceptibility and Resistance Pattern of Isolated Organisms

Table 7 displays the antibiotic sensitivity and resistance trends of the isolated microorganisms. Overall, 84% of the isolated organisms were resistant to the recommended first-line antibiotics for ampicillin and 71.3% for gentamicin. Cefotaxime had a resistance pattern of 78.1%, ceftriaxone had a resistance pattern of 76.2%, vancomycin had a resistance pattern of 35.9%, and amikacin had a resistance pattern of 17.5%. The *Staphylococcus* infections were shown to be 95.7% resistant to penicillin and 80.43% resistant to ampicillin. Klebsiella was resistant to ampicillin in 92.3% of cases, cefotaxime in 82.5% of cases, and gentamicin in 69.2% of cases. *E. coli* was resistant to gentamicin in 86.7% of cases, ampicillin in 75% of cases, and ceftriaxone in 93.7% of cases. *Acinetobacter* was completely resistant to ampicillin and 80% resistant to gentamicin. *Streptococcus* was completely resistant to gentamicin, completely resistant to penicillin G, and completely resistant to imipenem. *Proteus* was found to be completely resistant to ceftriaxone, completely resistant to ciprofloxacin, and completely resistant to aztreonam.

**Table 7:** Antibiotic susceptibility and resistance pattern of isolated organisms.

**Antibiotics**	**Category Number tested (%)**	**Gram-positive bacteria** ***N*** **(%)**		**Gram-negative bacteria** ***N*** **(%)**					
		***Staphylococcus aureus***	***Streptococcus* spp**.	***Klebsiella* spp**.	***E. coli***	***Pseudo-monas***	***Proteus* spp**.	***Serratia***	***Acinetobacter***
Penicillin G	Sensitive 3 (4.2)	3 (4.3)	0 (0)						
	Resistant 68 (95.8)	67 (95.7)	1 (100)						
Ampicillin	Sensitive 13 (16.0)	9 (19.6)	1 (100)	2 (7.7)	1 (25)	0 (0)			0 (0)
	Resistant 68 (84.0)	37 (80.4)	0 (0)	24 (92.3)	3 (75)	2 (100)			2 (100)
Amoxicillin–clavunate	Sensitive 57 (40.4)	29 (40.8)	1 (100)	20 (40.8)	4 (30.8)	1 (50)		1 (100)	1 (25)
	Resistant 84 (59.6)	42 (59.2)	0 (0)	29 (59.2)	9 (69.2)	1 (50)		0 (0)	3 (75)
Oxacillin	Sensitive 5 (15.2)	5 (15.6)	0 (0)						
	Resistant 28 (84.8)	27 (84.4)	1 (100)						
Cloxacillin	Sensitive 0 (0)		0 (0)						
	Resistant 1 (100)		1 (100)						
Piperacillin–tazobactam	Sensitive 22 (44.0)	4 (36.4)		11 (57.9)	3 (33.3)	3 (60)	1 (100)		0 (0)
	Resistant 28 (56.0)	7 (63.6)		8 (42.1)	6 (66.7)	2 (40)	0 (0)		5 (100)
Cefoxitin	Sensitive 19 (33.9)	18 (35.3)	1 (50)	0 (0)	0 (0)				
	Resistant 37 (66.1)	33 (64.7)	1 (50)	2 (100)	1 (100)				
Ceftriaxone	Sensitive 19 (23.8)	8 (50)		7 (17.1)	1 (6.3)	3 (60)	0 (0)	0 (0)	
	Resistant 61 (76.2)	8 (50)		34 (82.9)	15 (93.7)	2 (40)	1 (100)	1 (100)	
Cefotaxime	Sensitive 14 (21.9)	2 (100)		7 (17.5)	2 (16.7)	2 (40)			1 (20)
	Resistant 50 (78.1)	0 (0)		33 (82.5)	10 (83.3)	3 (60)			4 (80)
Ceftazidime	Sensitive 12 (23.1)	1 (25)		8 (22.9)	1 (14.3)	2 (33.3)			
	Resistant 40 (76.9)	3 (75)		27 (77.1)	6 (85.7)	4 (66.7)			
Aztreonam	Sensitive 15 (30.0)	2 (22.2)		10 (38.5)	1 (20)	2 (66.7)	0 (0)	0 (0)	0 (0)
	Resistant 35 (70.0)	7 (77.8)		16 (61.5)	4 (80)	1 (33.3)	1 (100)	1 (100)	5 (100)
Imipenem	Sensitive 83 (96.5)	42 (97.7)	0 (0)	28 (100)	8 (100)	2 (66.7)			3 (100)
	Resistant 3 (3.5)	1 (2.3)	1 (100)	0 (0)	0 (0)	1 (33.3)			0 (0)
Meropenem	Sensitive 59 (69.4)	15 (46.9)	1 (100)	27 (84.4)	12 (85.7)	1 (50)			3 (75)
	Resistant 26 (30.6)	17 (53.1)	0 (0)	5 (15.6)	2 (14.3)	1 (50)			1 (25)
Vancomycin	Sensitive 41 (64.1)	40 (64.5)	1 (50)						
	Resistant 23 (35.9)	22 (35.5)	1 (50)						
Clindamycin	Sensitive 59 (80.8)	58 (81.7)	1 (50)						
	Resistant 14 (19.2)	13 (18.3)	1 (50)						
Ciprofloxacin	Sensitive 72 (46.8)	29 (40.3)	0 (0)	26 (46.4)	14 (73.7)	2 (100)	0 (0)	1 (100)	0 (0)
	Resistant 82 (53.2)	43 (59.7)	1 (100)	30 (53.6)	5 (26.3)	0 (0)	1 (100)	0 (0)	2 (100)
Gentamycin	Sensitive 35 (28.7)	18 (31.6)	0 (0)	12 (30.8)	2 (13.3)	1 (33.3)	0 (0)	1 (100)	1 (20)
	Resistant 87 (71.3)	39 (68.4)	1 (100)	27 (69.2)	13 (86.7)	2 (66.7)	1 (100)	0 (0)	4 (80)
Amikacin	Sensitive 33 (82.5)			22 (95.7)	6 (50)	2 (100)	1 (100)		2 (100)
	Resistant 7 (17.5)			1 (4.3)	6 (50)	0 (0)	0 (0)		0 (0)
Sulfamethoxazole–trimethoprim	Sensitive 15 (16.3)	8 (16)	0 (0)	4 (14.8)	3 (27.3)				0 (0)
	Resistant 77 (83.7)	42 (84)	2 (100)	23 (85.2)	8 (72.7)				2 (100)
Erythromycin	Sensitive 5 (12.8)	5 (13.5)	0 (0)						
	Resistant 34 (87.2)	32 (86.5)	2 (100)						
Chloramphenicol	Sensitive 28 (63.6)	3 (60)		16 (72.7)	8 (72.7)	0 (0)			1 (25)
	Resistant 16 (36.4)	2 (40)		6 (27.3)	3 (27.3)	2 (100)			3 (75)

*Cells shaded in gray indicate the isolates that were not tested*.

*N, number of isolates tested*.

## Discussion

Prompt diagnosis and treatment are crucial in decreasing infant sepsis mortality and sequelae. As a result, it is crucial to identify neonates who are at risk of developing sepsis and, if sepsis develops, to identify the characteristics linked with a bad prognosis as soon as possible. The mortality rate of newborn sepsis varies in institutions and countries. According to this study, the mortality rate is 45.4%. This was significantly higher than a study conducted in the same hospital in 2012, which found 13.9% (12). This demonstrates that the death rate owing to neonatal sepsis has increased significantly at Muhimbili National Hospital. This increase could be attributed to rising antibiotic resistance. Kayange et al. found a death rate of 28.5% of infants with positive blood cultures in a 2010 study at Bugando Medical Center in Tanzania (13). Different and some similar rates have been found in other studies conducted in South China (9.5%) (14), Uganda (15.2%) (15), India (36%), Bhutan (20.5%) (16), Nigeria (32.2%) (14), Niger (38.24%) (17), Zambia (43%) (18), and Congo (21%) (19). The discrepancies in death rates between studies can be attributed to a variety of factors, including socioeconomic factors, geographical factors, equipment levels, and the efficacy of each hospital's preventative and therapeutic strategies (19).

Additionally, this study evaluated the major predictors of death in patients with culture-proven sepsis. The significant predicting factors of death for culture-proven sepsis were found to include a birth weight of <1.5 kg, early-onset sepsis, lethargy, vomiting, respiratory distress, hyperglycemia, sepsis with Gram-negative bacteria, and mechanical ventilation. This study indicates that low birth weight (<1.5 kg) is a risk factor for death (OR, 1.441) in neonates with sepsis. A similar finding has been reported in studies from different countries (17, 19, 20). Newborns with weight <1.5 kg had increased mortality, possibly due to impairments in humoral and cellular immunity as well as prolonged hospitalization, which raises the risk of nosocomial infection. Infection is a big concern, and it compounds the already bad outcome for a baby born prematurely (17). Similarly, an Indian study (14) revealed that lethargy (OR, 1.583) and hyperglycemia (OR, 1.669) are significant predictors of neonatal death. Respiratory distress (OR, 2.207), vomiting (OR, 1.705), and mechanical ventilation (OR, 2.206) were also associated with high neonatal mortality in this present study. TNF, IL-1, IL-6, and IL-8 have been shown to be elevated in individuals with acute respiratory distress syndrome and septic shock. These cytokines' levels in the blood may help determine sepsis severity and prognosis (21, 22).

According to this study, Gram-positive bacteria (50.4%) predominated in causing newborn sepsis as compared to Gram-negative bacteria (49.6%). In contrast, a 2012–2016 study in South China reported that Gram-negative bacteria (*n* = 371) outnumbered Gram-positive bacteria (*n* = 218, 35.2%) and fungi (*n* = 30, 4.8%) (23). However, Gram-negative sepsis (OR, 1.424) was found to be a significant predictor of neonatal sepsis mortality when compared to Gram-positive sepsis in this present study. Other research yielded similar outcomes (12, 24). A greater mortality rate could be related to significantly higher CRP and IL-6 levels in Gram-negative bacteremia than in Gram-positive bacteremia (24). There are evidences for two separate mechanisms by which Gram-negative bacteria generate systemic reactions. Bacteria enter the circulation *via* a normal or damaged epithelium, triggering systemic immunological responses (such as enhanced vascular permeability, leukocyte–endothelial adhesion, complement, and clotting pathways) that lead to multiorgan failure. Toxins or circulating microorganisms are not necessary as direct stimuli for intravascular inflammation, according to a second idea (25). In our study, *S. aureus* was the most common cause in both early-onset sepsis and late-onset sepsis, but the percentage was higher in LOS at 58.97%. It must be noted that this percentage included all neonates, either alive or dead, with positive blood cultures. In a study by Said et al. *Staphylococcus capitis*-related sepsis was identified as an independent risk factor for severe morbidity in low-birth-weight infants with late-onset sepsis (26). *Staphylococcus epidermidis* has also been reported as the most prevalent pathogenic bacterium species in the LOS group (27). In this study, it can be observed in Table 1 that the majority of these neonates with sepsis have an extremely low birth weight (<1.5 kg; 73 out of 238) and low birth weight (1.5–2.5 kg; 94 out of 238), for a total proportion of 70.2% neonates. Furthermore, it was discovered that preterm neonates predominated (151 out of 238), accounting for ~63.4% of the overall sample. The presence of *S. aureus* in LOS may also be because very-late-onset sepsis is frequently diagnosed in newborns with extremely low birth weight who are usually hospitalized for several weeks after birth. The intravascular catheters required for their care, prolonged antimicrobial drug exposure, the persistence of immature host defensive mechanisms, prematurity, and a prolonged stay in the neonatal intensive care unit (NICU) are the most prominent risk factors for their predisposition (28). These findings imply that differences in host responses and pathogenicity mechanisms of different pathogenic microbes should be considered in the treatment of bacteremia patients and that new antimicrobial counter-measures beyond standard antimicrobial drugs are urgently needed (24). The routine reporting of Gram stain reaction laboratory data could significantly improve newborn sepsis.

The EOS group had a death rate 55.9% greater than the LOS group (36.4%), indicating that factors such as maternal genitourinary tract infections directly affect the occurrence of infant sepsis. Ogunlesi and his colleague found comparable results (14). Nevertheless, LOS has also been linked to an elevated mortality rate in some studies (29, 30).

Overall, the isolated organisms were resistant to 84% of ampicillin and 71.3% of gentamicin. Ceftriaxone resistance was 76.2%, cefotaxime resistance was 78.1%, vancomycin resistance was 35.9%, and amikacin resistance was 17.5%. Inappropriate antibiotic use may be a contributing factor to the hospital's extremely high levels of antibiotic resistance documented in this and previous research (9, 31). Between 1999 and 2012, investigations conducted at MNH and Bugando in Tanzania (1, 10, 12–14, 32) showed a progressive increase in resistance not just to first-line antibiotics but also to alternative drugs such as cefotaxime, vancomycin, and amikacin. Surprisingly, in this investigation, we observed an alarming increase in resistance to ceftriaxone, gentamicin, and amikacin compared to previous studies conducted in the same setting in 2012 (10, 12) showing an unprecedented increase in antibiotic resistance. In our study, we discovered an alarmingly high level of vancomycin resistance. Out of 62 isolates tested for *S. aureus* sensitivity and resistance, 22 (35.5%) were resistant to vancomycin. Vancomycin has been the first-line treatment for methicillin-resistant *S. aureus* since its discovery (33). The emergence of vancomycin resistance poses a serious threat to public health around the world. A recent study (34) found that vancomycin-resistant *S. aureus* was recently discovered in Egyptian slaughterhouses. According to recent reports from Michigan, USA, *S. aureus* has decreased susceptibility and resistance to vancomycin in July 2021 (33). As a result, it is critical to monitor these infections and conduct additional research to determine why antimicrobial resistance is increasingly becoming a problem.

The strengths of this study include a single center for all investigations, ensuring that records and common practice were consistent; the laboratory analyses were done in one microbiology laboratory; and a relatively large and recent cohort of neonates with proven sepsis were included, making the obtained information for antimicrobial resistance and bacterial profiles relevant to the local population. However, because this is a retrospective study, it has some limitations. Because of the lack of intra-observer reliability, variable degrees of clinical skills/awareness in NICU settings, and incomplete documentation, retrospective chart reviews that attempt to capture the clinical features of neonatal sepsis are frequently incomplete. The procedure for collecting blood samples for cultures was also not documented in the files, and therefore we had to rely on information from the microbiology department about what the standard procedure should be. We were likewise unable to determine the precise timing of random blood glucose levels. It should be noted that there was no consistency in testing specific antibiotics against bacterial isolates at Muhimbili in this study, thus resulting in different susceptibility results.

## Conclusion and Recommendation

Infant demography research has found a significant connection between VLBW and early-onset sepsis as well as neonatal mortality in the newborn population. High blood sugar levels and mechanical ventilation were also identified as risk factors for neonatal mortality. As a result of neonatal sepsis' clinical manifestations, such as lethargy, vomiting, and respiratory distress, newborn mortality was found to be associated with these symptoms. Gram-negative bacteria are typically found in septicemic newborns who have died. Early detection and treatment of these risk factors would dramatically lower the likelihood of serious and life-threatening problems in newborns as well as the likelihood of mortality in these babies. In addition, this study discovered an abnormally high level of resistance to common antibiotics, which is of great concern. Antibiotic resistance may be due to overuse and misuse of antibiotics. We advocate for a change of currently prescribed antibiotics in our setting, the need to do a blood culture as soon as sepsis is suspected, and the need for a prospective study which we are currently undertaking to bridge the gaps found in this study and also provide updated data for policy.

## Data Availability Statement

The data analyzed in this study is subject to the following licenses/restrictions: These are retrospective datasets analyzed for the purposes of this study and can only be shared upon reasonable request and subsequent approval from the Ethics Committee. Requests to access these datasets should be directed to archangelmntim@yahoo.co.uk; nura_abd@yahoo.com.

## Ethics Statement

The studies involving human participants were reviewed and approved by Ethics Committee of Muhimbili National Hospital, Tanzania. Written informed consent from the participants' legal guardian/next of kin was not required to participate in this study in accordance with the national legislation and the institutional requirements.

## Author Contributions

NB-a contributed to the data collection, data analysis, and manuscript preparation. JA contributed to the manuscript preparation. MN contributed to the manuscript preparation and revision. RT contributed to the data analysis and manuscript preparation. MM and HN contributed to the data collection and data analysis. HJ contributed to the conceptualization, design, and manuscript preparation. All authors contributed to the article and approved the submitted version.

## Conflict of Interest

The authors declare that the research was conducted in the absence of any commercial or financial relationships that could be construed as a potential conflict of interest.

## Publisher's Note

All claims expressed in this article are solely those of the authors and do not necessarily represent those of their affiliated organizations, or those of the publisher, the editors and the reviewers. Any product that may be evaluated in this article, or claim that may be made by its manufacturer, is not guaranteed or endorsed by the publisher.
